# Evaluation of Childhood Allergy Risk Among Pregnant Women in a Tertiary Care Hospital in Thailand

**DOI:** 10.7759/cureus.63322

**Published:** 2024-06-27

**Authors:** Dittakarn Boriboonhirunsarn, Siraluck Puttapratimonk

**Affiliations:** 1 Obstetrics and Gynaecology, Faculty of Medicine Siriraj Hospital, Mahidol University, Bangkok, THA; 2 Obstetrics and Gynaecology, Wat Raja O Ros School, Bangkok, THA

**Keywords:** prevalence, knowledge, risk assessment, pregnancy, childhood allergy

## Abstract

Objectives: This study aims to evaluate the prevalence of pregnant women whose children are at higher risk for childhood allergies and to assess knowledge of risk assessment and prevention strategies.

Methods: A cross-sectional study was conducted on 310 pregnant women in an antenatal care clinic at a tertiary care hospital in Thailand. In addition to baseline demographic and obstetric characteristics, all participating pregnant women were asked to complete a questionnaire regarding risk evaluation and knowledge of childhood allergies on various topics. A childhood allergy risk assessment was evaluated based on the history of allergy disease in immediate family members. The questionnaire on knowledge was derived from a guideline issued by the Allergy, Asthma, and Immunology Association of Thailand, with possible scores of 0-30.

Results: The mean maternal age was 30.6 years, and 139 (44.8%) were nulliparous. Overall, 86 couples (27.7%) were at high risk for childhood allergies. The mean total knowledge score was 15.2 out of 30, and only 24 women (7.7%) had an overall score of >20, and 40 women (12.9%) had an overall score of ≤10. The mean knowledge score for almost every subtopic was less than half of the possible points, except for the risk reduction strategies during pregnancy. Comparisons between those with higher and lower scores (≥16 vs. ≤15 points) showed that women with higher knowledge scores were significantly more likely to have had a previous child with an allergy (p=0.010).

Conclusion: The prevalence of pregnant women whose children were at higher risk for childhood allergies was 27.7% (86 of 310 couples). The women had limited knowledge of childhood allergies with regard to risk assessment, risk reduction strategies, and various interventions. The only factor associated with a higher knowledge score was having a previous child with an allergy.

## Introduction

An increasing trend in allergies has been consistently reported worldwide. It has been estimated that major allergic conditions, including asthma, allergic rhinitis, atopic dermatitis, and food allergies, affect approximately 20% of the global population [1-3]. Childhood allergies can adversely affect not only their health but also other aspects, such as social, economic, quality of life, etc. [3,4]. In addition, it has been reported that children affected by any allergic condition are more likely to develop other allergic conditions in the future [5-7].

Various risk factors for childhood allergies have been reported, and the major risks were genetic, environmental, and food factors [6-11]. The results of previous studies showed that parental allergy has been shown to be an important risk for various childhood allergies, including asthma, allergic rhinitis, and atopic dermatitis, and maternal history of allergy might be more strongly related to childhood allergy than paternal history [10-14].

In Thailand, similar to other countries, the rapid changes in social, economic, environmental, and living conditions, as well as food consumption behavior, also result in increased exposure of children to various allergens early in their lives, which could increase their risks of allergy compared to the past. Previous studies showed that the prevalence of allergic rhinitis in children aged 6-7 years and 14 years was as high as 15% and 17.5%, respectively. Important risk factors were parental histories of allergies, including asthma, allergic rhinitis, and atopic dermatitis [15]. Another study also showed that the cumulative and 12-month period prevalence of common allergic conditions in children were 24.4% vs. 13.5% for wheezing, 51.1% vs. 43.6% for rhinitis, and 15.8% vs. 14.2% for eczema, respectively [16].

In 2020, the Allergy, Asthma, and Immunology Association of Thailand (AAIAT) issued a guideline for allergy prevention [17]. The topics in the guideline include a risk assessment checklist, a guide for risk reduction during pregnancy, delivery, and breastfeeding, a guide for the use of supplements by mothers and their newborns, the care of high-risk infants, the use of special infant formula, the use of emollients, and the household environment.

The guideline can be applied to the practice of caring for pregnant women throughout their pregnancy and postpartum period, which could help minimize the risk of allergies in their children in the future. However, the implementation has not been widely applied to pregnant women attending antenatal care. Therefore, the objectives of this study were to evaluate the prevalence of pregnant women whose children were at higher risk of childhood allergies and to assess knowledge of this specific issue. The results would provide more understanding of the risk of childhood allergies and would help improve the care of pregnant women in the future.

## Materials and methods

A cross-sectional study was conducted on pregnant women attending an antenatal care clinic at a tertiary care hospital after ethical approval from the Siriraj Institutional Review Board (approval number: 374/2023). Eligible pregnant women were asked if they were interested in participating in the study. Brief information about the study and related procedures was provided verbally along with the information sheet. The women were asked to sign an informed consent if they agreed to participate. A total of 310 low-risk, singleton pregnant women who agreed to participate were enrolled after informed consent was obtained. The sample size was estimated from a 20% prevalence of couples who were at higher risk for childhood allergies in a pilot study. At 95% confidence and 5% acceptable error, at least 275 women were required, including a 10% loss.

After baseline demographic and obstetric characteristics were recorded, all participating pregnant women were asked to complete a questionnaire regarding risk evaluation and knowledge of childhood allergies on various topics. The questionnaire was developed by the research team, and its content was derived from a guideline issued by the AAIAT [17]. The questionnaire included a childhood allergy risk assessment form, which was based on the history of allergy disease in immediate family members (mothers, fathers, and siblings), as shown in Table 1.

**Table 1:** Childhood allergy risk assessment form The risk assessment form was according to a guideline by the Allergy, Asthma, and Immunology Association of Thailand (AAIAT) [17]. ^a^ Major condition includes asthma, atopic dermatitis, allergic rhinitis, and cow’s milk allergy. ^b^ Minor conditions include urticaria, drug allergies, other food allergies, and allergic conjunctivitis. The total score was calculated from the sum of the highest scores for major and minor allergic conditions. The child was considered at higher risk for allergy if the total score was ≥2.

Family members	Types and severity of allergic conditions						Scores
	Major conditions ^a^			Minor conditions ^b^			
	Definite	Not sure	No	Definite	Not sure	No	
Father	2	1	0	1	0.5	0	
Mother	3	2	0	1	0.5	0	
Siblings	2	1	0	1	0.5	0	

The total score was calculated from the sum of the highest scores for major and minor allergic conditions. The child was considered at higher risk for allergy if the total score was ≥2. Knowledge of childhood allergy was also evaluated by another questionnaire, which was divided into seven subtopics: (1) risk assessment for childhood allergy (two items), (2) risk reduction strategies during pregnancy (six items), (3) nutritional supplements to reduce the risk of childhood allergy (four items), (4) care of high-risk infants to reduce the risk of allergy (seven items), (5) special infant formula to reduce the risk of childhood allergy (three items), (6) use of emollients (three items), and (7) household environment to reduce the risk of childhood allergy (five items). The contents of the questions were also based on the AAIAT recommendations. There were 11 negative questions. A score of 1 was given for each correct answer, and possible scores were between 0 and 30. The content of the questionnaire was validated by three experts in childhood allergies, and the questionnaire was initially tested for reliability with 30 pregnant women. The results showed that Cronbach’s alpha was 0.81.

After completing the questionnaire, all the women received information on the allergy risk of their children, and knowledge of appropriate care during pregnancy and newborn care was provided by the research team together with a paper-based summary of the AAIAT recommendations. This includes information on risk reduction strategies during pregnancy, appropriate nutritional supplements, care of high-risk infants, special infant formula, use of emollients, and household environment issues.

Descriptive characteristics were used to describe various characteristics, using mean, standard deviation, number, and percentage as appropriate. The prevalence of pregnant women whose childhood was at higher risk for childhood allergies was estimated. Total knowledge scores, scores of each subtopic, and each question were examined. The women were further divided into two groups according to their total knowledge score. Those with a score of more than half of the total scores (≥16 of 30) were considered to have adequate knowledge, while those with a score of ≤15 had limited knowledge. Various characteristics were compared between the two groups to determine possible associated factors. Continuous variables were compared using the student t-test, and categorical variables were compared using the chi-square test. A p-value of <0.05 was considered statistically significant.

## Results

A total of 310 pregnant women were included in the study. Baseline characteristics are shown in Table 2. The mean age was 30.6 years, the mean gestational age was 22.2 weeks of gestation, and 139 women (44.8%) were nulliparous. The majority worked as employees (164 cases, 52.9%), graduated with a bachelor's degree or higher (140 cases, 45.2%), and had adequate family income (254 cases, 81.9%).

**Table 2:** Baseline characteristics of pregnant women (N=310) GA: gestational age, SD: standard deviation

Characteristics	N (%)
Mean age ± SD (years)	30.6 ± 5.5
Mean GA ± SD (weeks)	22.2 ± 6.0
Nulliparous	139 (44.8)
Occupation	
Civil service	54 (17.4)
Employee	164 (52.9)
Business owner	51 (16.5)
Others	41 (13.2)
Education	
Secondary or lower	113 (36.5)
Vocational certificate	57 (18.4)
Bachelor's degree or higher	140 (45.2)
Nuclear family	137 (44.2)
Family income	
<30000 Baht	125 (40.3)
30000-50000 Baht	114 (36.8)
>50000 Baht	71 (22.9)
Adequate income	254 (81.9)

Table 3 shows the rates of couples at high risk for childhood allergies (risk score ≥2). Overall, 86 couples (27.7%) were at high risk. The risk score of ≥2 on the maternal side was found in 58 women (18.7%), while it was 23 on the paternal side (7.5%), and the risk was from a previous child in 20 cases (11.7%).

**Table 3:** Rate of couples at high risk for childhood allergies

Persons with risk score ≥2	N (%)
Mothers	58 (18.7)
Fathers	23 (7.5)
Previous child (n=171)	20 (11.7)
Overall risk score ≥2	86 (27.7)

Overall knowledge scores and scores for each subtopic are reported in Table 4. The mean total score was 15.2 of 30 possible points. The mean knowledge score for almost every subtopic was less than half of the possible points, except for the topic of risk reduction strategies during pregnancy. Further detailed analysis showed that only 24 women (7.7%) had an overall score of >20 and 40 women (12.9%) had an overall score of ≤10.

**Table 4:** Knowledge scores of pregnant women (N=310)

	Knowledge score
Overall score (mean ± SD)	15.2 ± 4.0
≤15	158 (50.9)
≥16	152 (49.1)
Knowledge topics	
Risk evaluation (2 items) (mean ± SD)	0.83 ± 0.64
Risk reduction during pregnancy (6 items) (mean ± SD)	3.35 ± 1.0
Use of supplements (4 items) (Mean ± SD)	1.08 ± 1.2
Care of high-risk infants (7 items) (mean ± SD)	3.91 ± 1.59
Use of special infant formula (3 items) (mean ± SD)	1.11 ± 0.79
Use of emollient (3 items) (mean ± SD)	0.8 ± 0.86
Household environment (5 items) (mean ± SD)	4.13 ± 0.8

Table 5 shows detailed information on the questions and answers. Only 149 women (48.1%) knew that the risk of childhood allergy increases if immediate family members are affected, and only 105 women (33.9%) knew that a child still has a chance of allergy even if no family members are affected. While the majority of the women correctly understood a balanced diet, the need to avoid common allergens, and smoking, 126 women (40.6%) thought that certain diets with a higher risk of allergy should be avoided, and 60 women (19.4%) thought cow’s milk consumption can cause cow’s milk allergy. Only 174 women (56.1%) thought or were not sure if cesarean delivery was not related to the risk of childhood allergies. Approximately one-third of the women correctly knew about probiotics and prebiotic supplementation during pregnancy and for high-risk infants to reduce the risk. In terms of appropriate care for high-risk infants, approximately two-thirds of the women knew about breastfeeding and food supplementation. As many as 206 women (66.5%) incorrectly thought that food allergy screening was recommended. Only half of the women knew that special infant formula can reduce the risk of childhood allergy when breast milk is inadequate and the formula is not better than breast milk. In addition, 107 women (34.5%) thought that other kinds of artificial milk could reduce the risk of childhood allergies. Approximately one-third of the women correctly knew about the use of emollients but thought that natural products were recommended. The majority of the women correctly knew about improving the household environment to reduce the risk of childhood allergies, but one-third thought that an air purifier was not necessary in closed rooms.

**Table 5:** Knowledge of various topics on childhood allergies * negative questions. Bold numbers indicate correct answers.

Questions	Yes N (%)	No N (%)	Not sure N (%)
Risk assessment for childhood allergies			
Allergies in immediate family members (mothers, fathers, brothers, sisters) increase the risk of childhood allergies.	149 (48.1)	81 (26.1)	80 (25.8)
There is no risk of childhood allergies if there is no risk for allergies in immediate family members (mothers, fathers, brothers, sisters). *	113 (36.5)	105 (33.9)	92 (29.6)
Risk reduction strategies during pregnancy			
A balanced diet is recommended.	298 (96.2)	6 (1.9)	6 (1.9)
Some diets at higher risk for allergies should be avoided, such as cow’s milk, eggs, soybeans, peanuts, wheat, seafood, etc. *	109 (35.2)	126 (40.6)	75 (24.2)
Pregnant women should avoid common allergens, such as dust mites, cockroaches, etc.	281 (90.6)	10 (3.2)	19 (6.2)
First- and second-hand smoking during pregnancy can increase the risk of childhood allergies.	236 (76.1)	24 (7.7)	50 (16.2)
Regular cow’s milk consumption can cause cow’s milk allergies in the child. *	152 (49.0)	60 (19.4)	98 (31.6)
Cesarean delivery is not related to the risk of childhood allergies. *	33 (10.6)	136 (43.9)	141 (45.5)
Nutritional supplements to reduce the risk of childhood allergies			
Probiotic supplementation during pregnancy can reduce the risk of childhood atopic dermatitis and food allergies.	103 (33.2)	30 (9.7)	177 (57.1)
Probiotic supplementation to infants at high risk can reduce the risk of childhood atopic dermatitis and food allergies.	98 (31.6)	26 (8.4)	186 (60.0)
Probiotics supplementation during pregnancy and to high-risk infants can reduce the risk of childhood asthma and allergic rhinitis. *	94 (30.4)	24 (7.7)	192 (61.9)
The use of some types of prebiotics can reduce the risk of some types of childhood allergies, such as cow’s milk allergies.	109 (35.2)	23 (7.4)	178 (57.4)
Care of high-risk infants to reduce the risk of allergies			
Infants should be breastfed for at least 4 months.	203 (65.5)	77 (24.8)	30 (9.7)
Food supplements can be initiated after the age of 2 months. *	46 (14.8)	185 (59.7)	9 (25.5)
For food supplements, they should be cooked and new supplements should be initiated every 3-5 days.	185 (59.7)	22 (7.1)	103 (33.2)
Food that can increase the risk of allergies, such as cow’s milk, soybean, whole eggs, wheat flour, etc., should be initiated after the age of 6 months.	197 (63.5)	25 (8.1)	88 (28.4)
All seafood should be initiated after the age of 1 year.	154 (49.7)	30 (9.7)	126 (40.6)
Infant formula can be provided if breast milk is inadequate.	260 (83.9)	18 (5.8)	32 (10.3)
A visit for food allergy screening is recommended before initiation of food supplements, even if there is no history or symptoms suspicious of food allergies. *	206 (66.5)	24 (7.7)	80 (25.8)
Special infant formula to reduce the risk of childhood allergies			
If breast milk is inadequate, special infant formula can reduce the risk of childhood allergies, such as atopic dermatitis and cow’s milk allergies during the first 6 years of age.	156 (50.3)	20 (6.5)	134 (43.2)
The use of soy milk, goat’s milk, or amino acid-based formula can reduce the risk of childhood allergies. *	107 (34.5)	29 (9.4)	174 (56.1)
Special infant formula is better than breast milk in reducing the risk of childhood allergies. *	36 (11.6)	158 (51.0)	116 (46.4)
Use of emollients			
The use of emollients in infants at high risk for allergies can reduce the risk of atopic dermatitis.	101 (32.6)	65 (21.0)	144 (46.4)
Emollients can be used from 1-3 weeks after birth to 6-8 months of age.	116 (37.4)	52 (16.8)	142 (45.8)
Emollients from natural products, such as milk, oatmeal, and wheat flour, are recommended. *	115 (37.1)	30 (9.7)	165 (53.2)
Household environment to reduce the risk of childhood allergies			
Keep household environment tidy and clean.	302 (97.4)	2 (0.6)	4 (1.3)
Beddings should be cleaned regularly and anti-dust mite bedsheets are recommended.	302 (97.4)	3 (1.0)	5 (1.6)
Pets are allowed but close contact should be avoided.	270 (87.1)	27 (8.7)	13 (4.2)
Remove the environment that attracts cockroaches or use traps to remove cockroaches.	294 (94.8)	4 (1.3)	12 (3.9)
An air purifier is not necessary in closed rooms. *	101 (32.6)	109 (35.2)	100 (32.2)

The women were divided into two groups based on knowledge scores, i.e., those with higher (≥16 points, n=152) and lower scores (≤15 points, n=158). Various characteristics were compared between the two groups, and the results are shown in Table 6. While most of the characteristics were comparable between the two groups, women with higher knowledge scores were significantly more likely to have had a previous child with an allergy (p=0.010).

**Table 6:** Comparison of characteristics between different knowledge scores

Characteristics	Knowledge score ≤15 N=158	Knowledge score ≥16 N=152	p-value
	N (%)	N (%)	
Mean age ± SD (years)	30.5 ± 5.5	30.8 ± 5.4	0.657
Nulliparous	78 (49.4)	61 (40.1)	0.102
Occupation			0.578
Civil service	23 (14.6)	31 (20.4)	
Employee	86 (54.4)	78 (51.3)	
Business owner	28 (17.7)	23 (15.1)	
Others	21 (13.3)	20 (13.2)	
Education			0.217
Secondary or lower	65 (41.1)	49 (31.6)	
Vocational certificate	27 (17.1)	30 (19.7)	
Bachelor's degree or higher	66 (41.9)	74 (48.7)	
Nuclear family	70 (44.3)	67 (44.1)	0.968
Family income			0.185
<30000 Baht	71 (44.9)	54 (35.5)	
30000-50000 Baht	56 (35.4)	58 (38.2)	
>50000 Baht	31 (19.6)	40 (26.3)	
Adequate income	123 (77.8)	131 (86.2)	0.057
At risk	45 (28.5)	41 (27.0)	0.767
Previous child status			0.010
No previous child	78 (49.4)	61 (40.1)	
Previous child without allergy	76 (48.1)	75 (49.3)	
Previous child with allergy	4 (2.5)	16 (10.6)	

## Discussion

The prevalence of allergic disease has shown an increasing trend in recent decades, and most affected cases were children [1,2,5,6]. Parental allergy has been consistently reported to be an important risk for childhood allergies, including asthma, allergic rhinitis, and atopic dermatitis [10-15,18]. The results of this study showed that the prevalence of pregnant women whose children were at high risk for childhood allergies was in 86 couples (27.7%). In a recent cross-sectional study in China, 1710 of 3437 children (49.75%) were likely to have allergic diseases based on the child's birth conditions and early life environment, the mother's lifestyle during pregnancy, and the father's [12].

The increased risk of childhood allergies has been reported to increase with a higher number of family members with allergies, as well as a higher number of allergic conditions [9-11,18]. Some studies also reported that maternal allergies, especially asthma, increase the risk of offspring disease to a greater extent than paternal diseases [5,11,14]. However, the results showed that only approximately half of the women knew that the risk of childhood allergies increased if immediate family members were affected.

In terms of knowledge, only half of the women had a knowledge score of more than half of the possible points, reflecting inadequate knowledge of this important issue. The mean knowledge score of almost every subtopic was also less than half of the possible points, except for the topic of risk reduction during pregnancy. However, 184 women (59.4%) misunderstood or were not sure that they needed to avoid a diet at higher risk for allergy, and only 60 women (19.4%) knew that cow’s milk consumption is not related to cow’s milk allergy in the child. A correct understanding of these issues needs to be provided to all pregnant women.

More than half of the women incorrectly thought or were not sure that cesarean delivery was not related to childhood allergies. Many pieces of evidence consistently suggest that cesarean sections are among the most important risks for childhood allergies, especially asthma and allergic rhinitis [19-22]. As the cesarean section rate continues to increase, the operation should be limited to those with medical indications, not only for the health of the mothers but the newborns as well. In addition, the risk of childhood allergies related to cesarean delivery should be communicated to every pregnant woman as part of preoperative counseling, especially among those performed without appropriate indications. Breastfeeding is strongly encouraged among children delivered by cesarean section, as it can attenuate the risk [19].

In terms of nutritional supplementation, the women still had limited knowledge of probiotic supplementation to reduce the risk of childhood allergies. Prenatal probiotics have been shown to have the potential to reduce infant allergies, particularly in children at high risk of allergy development [23]. A previous systematic review showed that probiotic supplementation during the third trimester of pregnancy and continued for the first three months of life reduces the incidence of atopic dermatitis in children. However, the addition of prebiotics did not appear to provide additional protection against the development of allergic disease in children [24]. A more recent systematic review reported that probiotic supplementation during pregnancy and infancy reduces food allergy risk and correlates with age-related changes in gut microbial composition in children [25]. Therefore, supplementation with probiotics should be recommended for high-risk mothers and newborns in order to reduce the risk of childhood allergies.

Although there is conflicting evidence [26], some types of special infant formulas may have the potential to reduce the risk of childhood allergies, especially atopic dermatitis and cow’s milk allergies, which are partially hydrolyzed whey and extensively hydrolyzed casein formulas [17]. However, this is only recommended for high-risk children when breast milk is inadequate. This issue was correctly understood by only half of the women, and, surprisingly, 158 women (51%) thought that these special infant formulas were better than breast milk in reducing the risk of childhood allergy.

Emollients have been shown to reduce the incidence of atopic dermatitis in the first year of life in high-risk infants [27-29]. However, only one-third of the women in this study correctly understood, and most of the women were not sure about the benefits of emollients.

The only factor associated with a higher knowledge score was having a previous child with an allergy. This might be because these parents had more experiences and received more information on the issue of caring for their affected children. Therefore, if this information were provided to the women during pregnancy, they would have more knowledge and could modify their practices to reduce the risk of childhood allergies.

The strength of this study was that it is probably among a few studies that examine this important issue among pregnant women and probably the first in Thailand. The risk assessment scores and knowledge questionnaire were adopted from the guidelines issued by the AAIAT [17], which makes them valid and reliable. The limitations might include that the history of allergies in the family was self-reported, which might not be totally valid. However, all the women were informed to report their family history of allergy when the conditions were diagnosed by doctors, not only from their perceptions, which could minimize the possibility of information bias. However, some biases might still exist. In addition, the study was conducted in a single tertiary care hospital; thus, generalization of the results might be limited due to differences in population and risks. Nationwide or context-specific studies are warranted to provide more information and generalizability. The nature of a cross-sectional study also limits the ability to establish causality between the variables assessed. In addition, some confounding factors might exist and could potentially influence the results. The study also lacks follow-up data to assess the long-term impact of the educational intervention on the knowledge and behavior of the women. Further studies are recommended to explore this important issue in more detail.

As the risk evaluation was quite simple, all pregnant women should be assessed for the risk as part of their antenatal care routine. Important information on the interventions to reduce the risk should be provided, especially to at-risk women. Moreover, some common misunderstandings should be clarified. This could help raise awareness and possibly reduce the occurrence of childhood allergies in the future.

## Conclusions

The prevalence of pregnant women whose children were at higher risk for childhood allergies was 27.7%. The women had limited knowledge of childhood allergies with regard to risk assessment, risk reduction strategies, and various interventions. The only factor associated with a higher knowledge score was having a previous child with an allergy.
